# A yeast-optimized single-cell transcriptomics platform elucidates how mycophenolic acid and guanine alter global mRNA levels

**DOI:** 10.1038/s42003-021-02320-w

**Published:** 2021-06-30

**Authors:** Guste Urbonaite, Jimmy Tsz Hang Lee, Ping Liu, Guillermo E. Parada, Martin Hemberg, Murat Acar

**Affiliations:** 1 Systems Biology Institute, Yale University, West Haven, CT USA; 2 Department of Molecular Cellular and Developmental Biology, Yale University, New Haven, CT USA; 3 Wellcome Sanger Institute, Wellcome Genome Campus, Hinxton, UK; 4 Department of Physics, Yale University, New Haven, CT USA; 5 Present Address: Evergrande Center for Immunologic Disease, Harvard Medical School and Brigham and Women’s Hospital, Boston, MA USA

**Keywords:** Gene expression analysis, Saccharomyces cerevisiae, Biochemical networks, Genetic interaction

## Abstract

Stochastic gene expression leads to inherent variability in expression outcomes even in isogenic single-celled organisms grown in the same environment. The Drop-Seq technology facilitates transcriptomic studies of individual mammalian cells, and it has had transformative effects on the characterization of cell identity and function based on single-cell transcript counts. However, application of this technology to organisms with different cell size and morphology characteristics has been challenging. Here we present yeastDrop-Seq, a yeast-optimized platform for quantifying the number of distinct mRNA molecules in a cell-specific manner in individual yeast cells. Using yeastDrop-Seq, we measured the transcriptomic impact of the lifespan-extending compound mycophenolic acid and its epistatic agent guanine. Each treatment condition had a distinct transcriptomic footprint on isogenic yeast cells as indicated by distinct clustering with clear separations among the different groups. The yeastDrop-Seq platform facilitates transcriptomic profiling of yeast cells for basic science and biotechnology applications.

## Abstract (web summary)

Guste Urbonaite and Jimmy Tsz Hang Lee et al. present a droplet-based single-cell sequencing method optimized for use in yeast. They use their method—yeastDropSeq—to investigate the changes in gene expression profiles following treatment with the lifespan-extending compound mycophenolic acid and/or its epistatic agent, guanine.

## Introduction

**Fig. 1:**
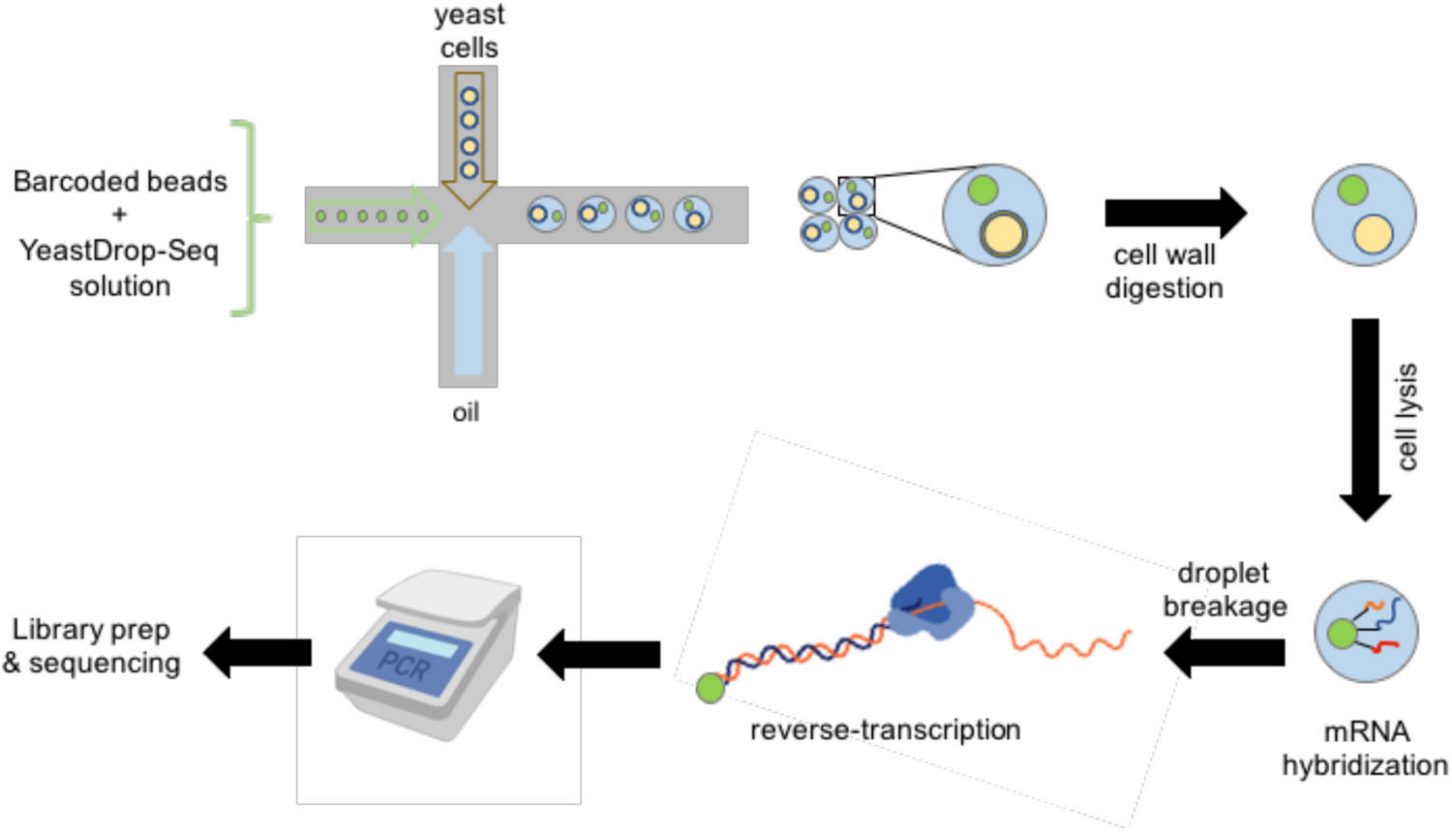
A schematic of the single-cell mRNA sequencing workflow for transcriptomic analysis of yeast cells. The yeastDrop-Seq solution containing chemicals for cell-wall digestion and cell lysis is mixed with barcoded microbeads, and the mixture is flown through one inlet of a microfluidic device. Yeast cells with intact cell walls and oil are flown through the second and third inlet of the microfluidic device, respectively. Encapsulated cells are then incubated to allow for cell-wall breakage and lysis. mRNA molecules hybridize to the barcoded microbeads via their polyA tails, droplets are broken, and reverse transcription occurs. cDNA is PCR-amplified and the cDNA libraries are prepared and submitted for sequencing.

In recent years, single-cell RNA sequencing (scRNA-seq) has become a widely used method for studying the transcriptomes of individual cells^1^. scRNA-seq has been used to identify populations of specific cell types within tissues^2^, to study particular cellular pathways^3^, and to better understand disease development^3^. One method to perform scRNA-seq is the Droplet Sequencing (Drop-Seq) platform^2^. Using a microfluidic device, this platform allows for the encapsulation of single cells, along with microbeads carrying barcoded primers and lysis buffer into oil droplets (Fig. 1). Upon cell lysis in the oil droplet, individual mRNA molecules hybridize to the primers on the microbeads. The barcoded primer sequences on each microbead allow for the distinction of mRNAs obtained from different cells and unique mRNAs obtained from the same cell. Next, all droplets are broken together and release their hybridized beads. Isolated beads are reverse-transcribed with template switching, generating cDNA strands. This is followed by the PCR-amplification of the cDNAs and the addition of the sequencing adapters. Finally, the barcoded mRNA samples are sent out for sequencing.

The original Drop-Seq platform has been specifically developed and optimized for mammalian cells with the goal of measuring mRNA counts at the single-cell level across genetic backgrounds and/or growth conditions. Size and structure/morphology differences between different cell types have made it difficult to directly apply the mammalian Drop-Seq platform to other organisms, including yeast which are much smaller than mammalian cells and have a cell wall. While there are well-based^4^, FISH-based^5^ and droplet-based^6^ (using commercial 10x Genomics platform) methods for measuring single-cell mRNA abundances in yeast cells, a table-top noncommercial Drop-Seq-based platform optimized for robust measurements of mRNA counts in yeast has not been available.

Here we present the yeastDrop-Seq platform, a yeast-optimized table-top scRNA-seq technology based on the original Drop-Seq platform^2^. As a proof-of-principle application of yeastDrop-Seq, we measure how Mycophenolic acid (MPA) and guanine impact mRNA counts globally at the single-cell level. MPA is a lifespan-extending compound that decreases de novo GMP synthesis^7–9^. Our work uncovers the global transcriptomic effects of MPA and guanine in a pathway-specific manner at single-cell resolution, providing novel insights about how MPA extends the lifespan in yeast.

## Results

### yeastDrop-Seq as a yeast-optimized scRNA-Seq method

We modified the original Drop-Seq protocol so that the differences in size and morphology between yeast and mammalian cells are factored in the relevant steps of the protocol.

To make sure that only one cell is present in each oil droplet, cell density at the time of cell-feeding into the microfluidic chip had to be sufficiently low. On the other hand, a density that is too low would lead to unfeasibly long cell-feeding times. We found that a density of 50 cells per microliter addressed these needs.

Unlike mammalian cells, yeast has a cell wall surrounding its outer membrane. To break the cell wall, we designed a solution containing Zymolyase in addition to a cell-lysis reagent and SDS (Methods). Zymolyase is a cell-wall digestion enzyme which does not alter RNA stability or folding^10^. After the initial formation of oil droplets by feeding into the microfluidic chip yeast cells (at the density of 50 cells per microliter) together with the microbeads resuspended in this solution, the oil droplets were incubated for 30 min at 30 °C to ensure that Zymolyase breaks the cell walls and then cell lysis occurs (Supplementary Fig. 1). After this incubation period, we evaluated oil-droplet quality and found that more than 95% of the droplets remained intact.

Next, oil droplets were broken, and the original Drop-Seq protocol^2^ steps were applied to the pooled mRNA molecules hybridized to the primers on the microbeads, including reverse transcription, exonuclease I treatment, PCR-amplification of the cDNAs and the addition of the sequencing adapters. Sequencing was performed using the Illumina HiSeq2500 platform with 2 × 100 nts read pairs.

### Measuring the single-cell transcriptomic impact of MPA and guanine

In our previous studies, we have elucidated that Mycophenolic acid (MPA) extended lifespan in yeast through GMP synthesis inhibition^8,9^. We further found that the longevity effect of MPA was prevented by the supplementation of exogenous guanine, MPA’s epistatic agent in GMP synthesis. MPA targets GMP synthesis by inhibiting IMP Dehydrogenase (IMD), decreasing de novo GMP synthesis^7–9^. IMD catalyzes the production of xanthosine 5’-phosphate (XMP) from inosine 5’-phosphate (IMP). This is followed by the production of GMP from XMP through the action of the GMP synthase Gua1. The guanylate kinase Guk1 converts GMP to GDP^7^. Despite the biochemical knowledge about MPA’s involvement in the GMP synthesis pathway, how it affects single-cell transcriptomes downstream of GMP/GDP/GTP synthesis has been unknown.

To study the global impact of MPA and guanine on single-cell transcriptomes and to identify the specific genes that are up/downregulated upon MPA and/or guanine treatment, we grew yeast cells for 18 h in media containing DMSO (D), MPA (M), Guanine (G), or MPA + Guanine (MG) (Methods), subjected the cells to yeastDrop-Seq, and processed the raw mRNA sequencing data to obtain single-cell level mRNA abundances.

The processing of the raw data included trimming low quality reads in sequences (Supplementary Fig. 2), mapping the resulting reads to the *S. cerevisiae* genome, and the construction of [gene × cell] expression matrices for each treatment condition. We next performed quality control of the processed data to filter out low quality cells and features in order to improve the signal-to-noise ratio for downstream analyses. For this, thresholds were adjusted according to the total Unique Molecular Identifier (UMI) counts and total gene counts (Supplementary Fig. 3); cells whose total UMI or feature counts fell outside of the normal distribution were excluded.

### Analysis of gene expression datasets for the rate of cell-doublet formation

An essential aspect of a droplet-based single-cell RNA measurement platform is its ability to encapsulate only one cell in each oil droplet or a low rate for doublet formation. To quantitatively characterize the doublet rate associated with yeastDrop-Seq, we applied five methods: DoubletFinder, Scrublet, DoubletDecon, scds, and solo (Supplementary Fig. 4). These methods first introduce artificial doublets based on the original dataset, followed by the training of a classifier to distinguish singlets from doublets. The methods then provide a ranking/score for the cells from the original dataset, and a threshold for doublet identification is defined.

While DoubletDecon predicted a surprisingly high doublet rate, which we attribute to false positives, the other four methods predicted low (~2–7%) rates for potential doublets for experiments run at 50 cells/uL (Methods). Also, the number of conserved doublets (i.e., doublets with the same barcode reported by more than one method) identified by the four methods was 12 out of 844 (Supplementary Fig. 4). We conclude that the cell-doublet rate of the yeastDrop-Seq platform is reasonably low. As a comparison, the original Drop-Seq platform developed for mammalian cells reported cell-doublet rates up to 11.3% at cell concentrations of 100 cells/uL and less than 5% at cell concentrations of 50 cells/uL.

### Characterization of sequencing reads, and transcript and gene counts

We analyzed the filtered sequencing reads to obtain the condition-specific distributions of their GC contents and read lengths. We found that the distribution of GC contents of all valid reads in all valid cells peaks at around 23% for each of the four growth conditions, which matched the peak expected based on theoretical grounds (Supplementary Fig. 5a). From the distributions of read lengths, we found that the peak corresponded to 52–53 bp consistently for each condition (Supplementary Fig. 5b). The distribution of UMI counts showed that there was no bias toward transcript length (Supplementary Fig. 6a) and GC percentage (Supplementary Fig. 6b).

We next quantified the number of distinct mRNA transcripts, and identified the genes they corresponded to. First, analyzing our single-cell mRNA sequencing data for effective reads from valid barcodes (Supplementary Fig. 3) (Methods), we saw that the average number of effective reads from valid barcodes varied between 49.3% and 65.7% across the four growth conditions used. We then obtained the distribution of the number of distinct mRNA transcripts per cell for each treatment condition (Supplementary Fig. 3), and calculated the average number of distinct mRNA molecules (Supplementary Data 1). With the total number of cells identified in each condition ranging from 85 to 268, the mean transcript counts per cell ranged from 840.19 to 1335.62 across the four treatment conditions. Coefficients of variation (CV) were calculated across the cells in each treatment condition (Supplementary Data 2). We saw that the average number of distinct genes across cells of a sample, with the number of distinct genes quantifying the number of genes captured per cell in each sample, were 443 (G), 554 (M), 578 (MG), and 619 (D) (Supplementary Data 1). Since *S. cerevisiae* has 6275 genes, and since the set of distinct genes captured in each cell is different, the fraction of overall distinct genes captured by yeastDrop-Seq across all cells were 38.6% (M), 46.5% (G), 55.7% (D), and 62.2% (MG).

We also sorted our gene expression matrices for each gene represented across cells in each growth condition. Supplementary Data 2 shows the average single-cell mRNA transcript numbers averaged across all cells of each treatment condition, as well as the standard deviation (SD). To quantify transcriptional noise in the expression of each gene, we used CV as a metric and saw similar dependencies between CV and mean across the four treatment conditions. A second-degree polynomial function fitted the distribution of single-cell CV vs. mean transcript numbers pooled from all genes represented for each condition (Supplementary Fig. 7). As expected, genes with low transcript numbers were associated with high CV values.

### Isogenic yeast populations display distinct transcriptomic substructures

**Fig. 2:**
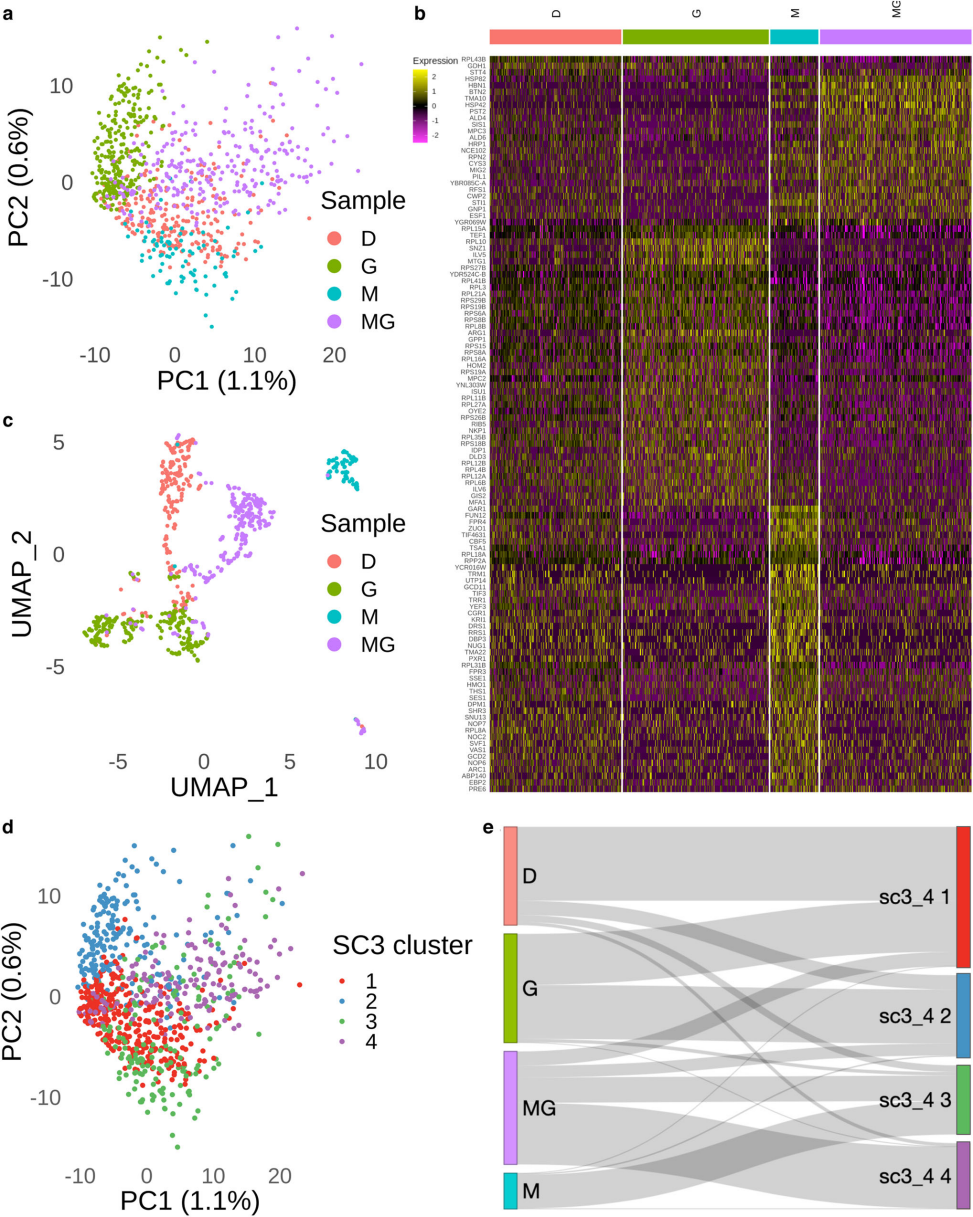
Identification of DE genes in the 4 samples. **a** PCA plot illustrating the distribution of the 4 samples (D *n* = 233, G *n* = 258, M *n* = 85, MG *n* = 268 cells). **b** Heatmap illustrating the DE genes corresponding to the 4 samples (Supplementary Data 3). The color represents the normalized expression level in natural-log scale of the corresponding genes of a cell (yellow is high). **c** UMAP plot of the 4 samples (D *n* = 233, G *n* = 258, M *n* = 85, MG *n* = 268 cells) based on the PC spaces in panel **a**. **d** Semi-supervised clustering identifies 4 clusters across the 4 samples. **e** Sankey plot illustrating the 4 samples mapping to the 4 SC3 clusters (cluster 1 *n* = 331, cluster 2 *n* = 193, cluster 3 *n* = 164, cluster 4 *n* = 156 cells). DE genes means differentially expressed genes.

To perform downstream analysis, we merged the data from the 4 samples, corresponding to the 4 growth conditions, using the ‘merge’ function in Seurat v3^11^, followed by normalization of the count matrices of the 4 samples. We performed Principal Component Analysis (PCA) on the merged gene expression data corresponding to the four treatment conditions (Fig. 2a). Each condition led to distinct clustering with clear separations among the different groups, which indicates that each treatment condition has a distinct transcriptomic footprint (Fig. 2b, Supplementary Data 3). The nonlinear dimension reduction performed through Uniform Manifold Approximation and Projection (UMAP) also showed consistent results (Fig. 2c). Despite the use of isogenic yeast cells across our experiments, we identified subclusters in the UMAP plot corresponding to transcriptomic heterogeneity within the DMSO, Guanine and MPA + Guanine-treated yeast populations; differently from these observations, the MPA treatment led to a relatively homogeneous population (Fig. 2c).

Although the PCA plot (Fig. 2a) clearly showed the presence of 4 clusters corresponding to the 4 treatment conditions, we wanted to obtain the best quantitative clustering outcome corresponding to our merged data. For this, we ran the single-cell consensus clustering (SC3) method which provides robust and accurate cell clustering as well as downstream analysis for single-cell RNA sequencing data^12^. We found that SC3 with k = 4 provided the best outcome for our combined data (Fig. 2d).

Using a Sankey diagram, we traced how results from each treatment condition were mapped to the four SC3 clusters (Fig. 2e). As expected due to the low population heterogeneity observed, almost all MPA-treated cells were mapped to a single SC3 cluster (SC3_4 3). On the other hand, most cells treated with DMSO or Guanine were mapped to three or four SC3 clusters.

**Fig. 3:**
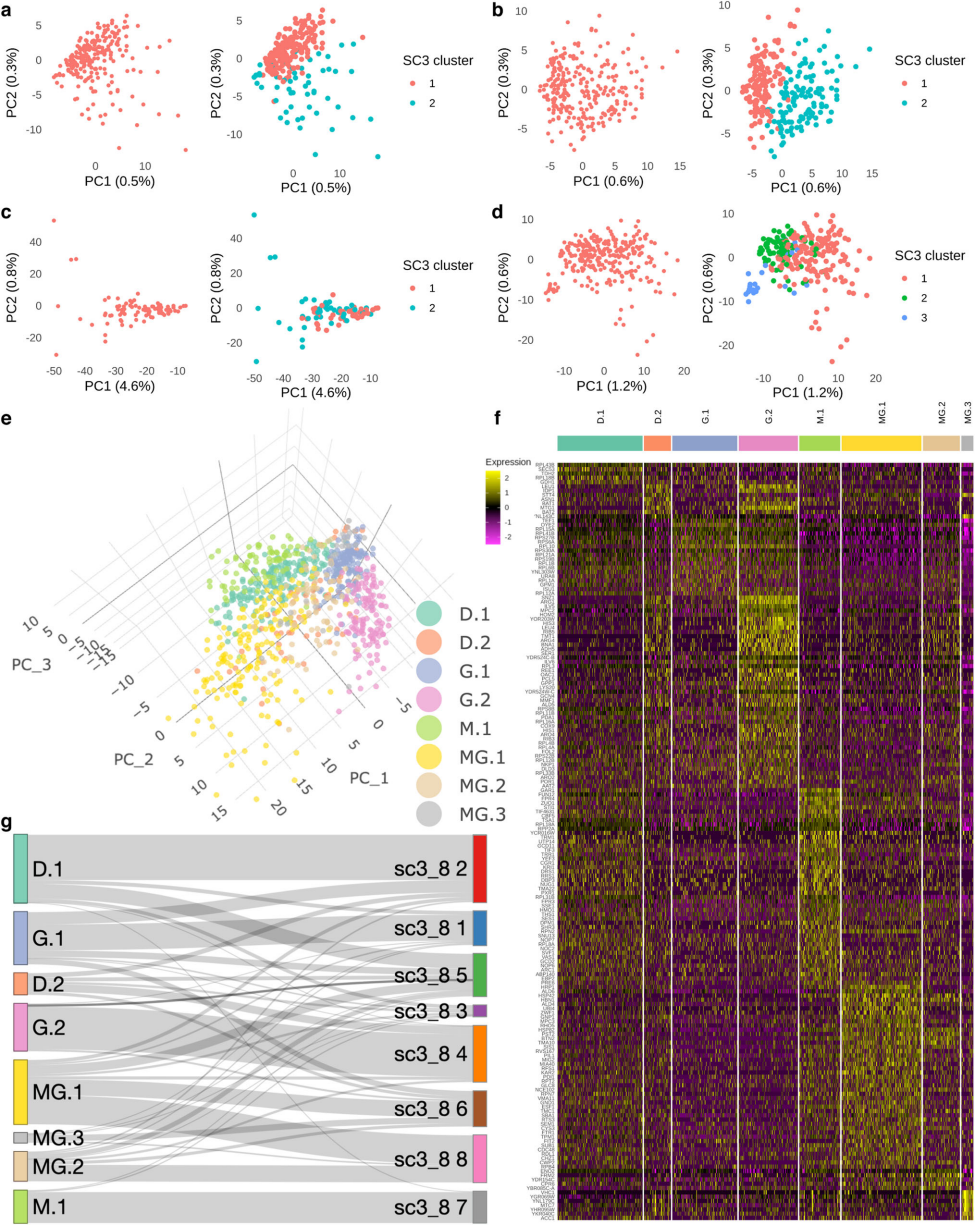
SC3 consensus clustering reveals the heterogeneity within yeast cells in the 4 samples. **a** PCA plot showing the 2 SC3 clusters in DMSO (*n* = 233 cells). **b** PCA plot showing the 2 SC3 clusters in Guanine (*n* = 258 cells). **c** PCA plot showing the 2 SC3 clusters in MPA (*n* = 85 cells). **d** PCA plot showing the 3 SC3 clusters in MPA + Guanine (MG *n* = 268 cells). **e** A 3-dimension PCA plot illustrating the distribution of the 8 subclusters (D.1 *n* = 177, D.2 *n* = 56, G.1 *n* = 135, G.2 *n* = 123, M.1 *n* = 85, MG.1 *n* = 166, MG.2 *n* = 77, MG.3 *n* = 25 cells) in the 4 samples. **f** Heatmap illustrating the DE genes corresponding to the 8 subclusters identified by SC3 (Supplementary Data 5). The color represents the normalized expression level in natural-log scale of the corresponding genes of a cell (yellow is high). **g** Sankey plot illustrating the subclusters identified in separated analysis mapping to the 8 SC3 clusters identified in group analysis. DE genes means differentially expressed genes.

To quantitatively evaluate the subpopulations associated with each treatment condition, we performed SC3 clustering analysis on each treatment-specific gene expression matrix separately. For the DMSO treatment, we found that k = 2 gave a reasonable resolution to distinguish two subclusters containing 177 and 56 cells (Fig. 3a). An area-under-the-receiver-operating-characteristic (AUROC) curve analysis identified the list of marker genes corresponding to each subcluster from the DMSO population (Supplementary Data 4). For the Guanine-treated cells, we again found two distinct subclusters represented by 135 and 123 cells (Fig. 3b). An AUROC curve analysis identified the list of marker genes corresponding to these subclusters (Supplementary Data 4). As expected for the MPA-treated cells, the SC3 analysis yielded a similar result: the apparent lack of heterogeneity prevented SC3 from resolving the population into more than one subcluster (Fig. 3c). Finally, for the cells treated with MPA and Guanine, there were 3 distinct subclusters (with 166, 77, 25 cells) and the result from consensus clustering showed that k = 3 was ideal (Fig. 3d), even though there were relatively small numbers of cells in the third subcluster. The small cluster was supported by the marker genes analysis which revealed a strong signal for the third subcluster (Supplementary Data 4).

Relabeling the cells with respect to the SC3 clustering results obtained from the analysis on each of the four treatment conditions, one can see the eight distinct subclusters under different plotting schemes (Fig. 3e, f, Supplementary Data 5). Finally, comparing these eight subclusters identified separately with the results obtained from a new SC3 clustering analysis (with k = 8) performed on the full data merged from all four treatment conditions, we found that a large fraction of cells could be consistently mapped between single subcluster counterparts (Fig. 3g).

### Identification of differentially expressed genes across treatment conditions and clusters

**Fig. 4:**
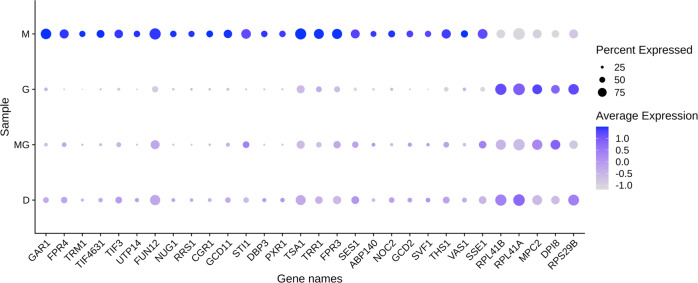
DE genes in the 4 samples. Dotplot showing the key DE genes (Methods) identified in the 4 samples (D *n* = 233, G *n* = 258, M *n* = 85, MG *n* = 268 cells). The size of the dot represents the percentage of cells within a sample expressing the corresponding genes, while the color represents the average expression level in natural-log scale of the corresponding genes across all cells within a sample (blue is high). DE genes means differentially expressed genes.

**Fig. 5:**
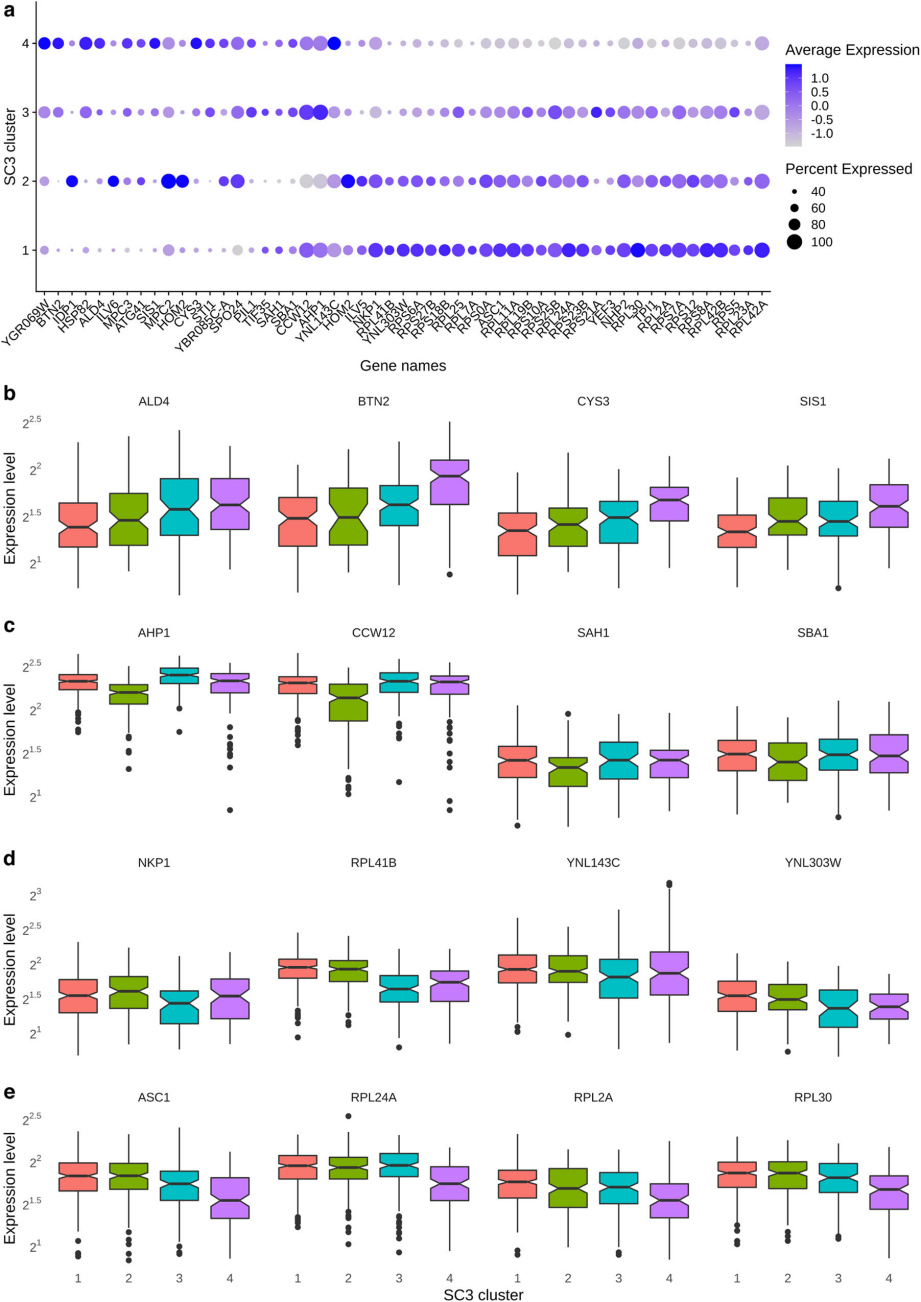
DE genes in the SC3 clusters with k = 4. **a** Dotplot showing the key DE genes (Methods) identified in the 4 SC3 clusters. The size of the dot represents the percentage of cells within a SC3 cluster expressing the corresponding genes, while the color represents the average expression level in natural-log scale of the corresponding genes across all cells within a sample (blue is high). **b** Boxplot showing the top 4 DE genes in SC3 cluster 1. **c** Boxplot showing the top 4 DE genes in SC3 cluster 2. **d** Boxplot showing the top 4 DE genes in SC3 cluster 3. **e** Boxplot showing the top 4 DE genes in SC3 cluster 4. Boxplots show the median (center line), interquartile range (hinges) and 1.5 times the interquartile range (whiskers); outlier data beyond this range are plotted as individual points. Expression levels are plotted in log 2 scale of normalized UMI (Methods). DE genes means differentially expressed genes. (cluster 1 *n* = 331, cluster 2 *n* = 193, cluster 3 *n* = 164, cluster 4 *n* = 156 cells).

We performed the Wilcoxon test to identify the differentially expressed genes. Using the thresholds of 50% of minimum percentage of cells in each condition/cluster and ±0.5 average log-fold change (with adjusted *p*-value < 0.05), we obtained a list of genes up- or downregulated for each treatment condition and cluster (Supplementary Data 6). Visual inspection of the heatmaps suggested that these genes could well represent each condition or distinct cluster (Fig. 3f). We also identified the gene candidates representing each subcluster in each treatment condition (Fig. 4, Supplementary Data 7). Finally, we obtained the set of differentially expressed genes for the clusters identified by SC3 with k = 4 (Fig. 5, Supplementary Data 8).

Performing a GO term analysis using the R package clusterProfiler^13^ we also found the pathways that are up- and downregulated in each treatment condition or treatment clusters. In Supplementary Data 9, we show the up- or downregulated pathways for each treatment condition. Delving deeper into the individual clusters for each treatment condition below we again identify up- or downregulated pathways or biological processes using the GO term analysis (Supplementary Data 10).

### Analysis of selected differentially expressed genes and biological processes in MPA-treated cells

We saw an upregulation of mRNAs for proteins involved in rRNA pre-processing in cells treated with MPA (*GAR1, UTP14, CGR1, DBP3*, and *PXR1*) (Fig. 4). However, it is known that MPA decreases ribosome biogenesis as well as rRNA levels, by decreasing RNA pol I and RNA pol III activity^14,15^. It has been suggested that this rRNA decrease leads to the accumulation of ribosomal proteins (r-proteins) in the cell^15^. We hypothesize that cells upregulate the processome machinery, transcribed by RNA pol II, in an attempt to produce more mature rRNA, as there is a lack of such rRNA upon treatment with MPA.

There is also an upregulation of tRNA synthetase and tRNA methyltransferase genes (*TRM1, SES1, ABP140, THS1, VAS1*) (Fig. 4). The decreased RNA PolI and PolIII activity due to MPA treatment has been shown to result in a decreased concentration of mature tRNAs^16^. We conjecture that MPA-treated cells attempt to replenish this population of tRNAs by upregulating genes, transcribed by RNA PolII, that are involved in their production. Histone and nucleosome assembly genes are also upregulated in cells treated with MPA (*FPR4, FPR3*) (Fig. 4). It is known that MPA acts as a transcription elongation repressor^17^, and we conjecture that MPA represses transcription by leading to a more packed chromatin structure. It is interesting to note that *FPR3* and *FPR4* are involved with rDNA, whose replication and recombination has been implicated in aging^18^. Additionally, translation initiation factor gene expression is upregulated, including *TIF3, GCD11, TIF4631, FUN12*, and *GCD2* (Fig. 4). We hypothesize that, because MPA treatment was previously reported to lead to a decrease in guanosine nucleotides^19^, protein production is decreased (rRNA and mRNA expression decreases) and cells attempt to rescue this phenotype by increasing translation initiation. Moreover, it was found that some components of the SAGA complex, which is involved in histone acetylation, translation initiation, and elongation, are also affected in cells treated with MPA, suggesting that MPA has an overall effect on the translation process^20^. MPA-treated cells also display an upregulation of antioxidant genes such as *TSA1* and *TRR1*. Cells treated with MPA appear to upregulate genes that provide protection from oxidative damage. This antioxidant effect of MPA has also been reported in mouse models previously^21^, and here we identify which yeast genes are involved in this process. Here we also observe that cells treated with MPA upregulate the expression of chaperone proteins (*STI1, SSE1*) (Fig. 4) that are involved in regulation of organization of amyloid-like proteins or unfolded proteins in general. It is interesting to note that cells treated with MPA have increased protein burden^22^. It is likely that upregulation of *STI1* and *SSE1* is a direct cause of this increased protein burden.

In MPA-treated cells, we saw an upregulation of genes, including *NUG1, NOC2*, and *RRS1* (Fig. 4), involved in ribosome component transport. It is interesting to note that cells treated with MPA also exhibited altered expression of 40 S and 60 S ribosomal subunits. Some subunit components are downregulated (*RPL41A, RPL41B*, and *RPS29B*) (Fig. 4), while others are upregulated (*RPL18A*). There is a delicate balance among the r-proteins forming the various subunits of the ribosome in the cell and it has been suggested that during ribosomal stress there can be an accumulation of free r-proteins not assembling in a ribosome^15^.

Additionally, cells treated with MPA downregulate mitochondrial components (*MPC2, DPI8*) (Fig. 4) and most likely mitochondrial function. It is known that mitochondrial membrane potential^23^ and mitochondrial composition^19^ are impacted by MPA treatment, so these downregulations upon MPA treatment, perhaps indicating a decrease in mitochondrial function, are not surprising.

Based on the GO terms analysis (Supplementary Data 9-10), there was an upregulation of ncRNA metabolic processes, involving tRNA aminoacylation for protein translation, as well as an upregulation of cellular component biogenesis involving proteasome components and ribosome assembly, such as amino acid activation, peptide biosynthetic process, translational initiation.

GO term analysis (Supplementary Data 9–10) also revealed several downregulated cellular processes including ribosomal RNA transport, ribosome assembly and ribosomal RNA metabolic process. In addition, mitochondrial function is also signified by a downregulation in pyruvate metabolic process. Additionally, phosphatidylinositol-mediated signaling was downregulated in MPA-treated cells. This result also made sense based on the previous finding that MPA alters cholesterol and phosphatidylcholine concentrations which greatly impacts lipid-mediated signaling in intestinal cells^19^. Regulation of cell wall organization was also downregulated in MPA-treated cells. Finally, we observed a downregulation in the monocarboxylic acid metabolic process in cells treated with MPA. As MPA itself is a monocarboxylic acid and perturbs many cellular pathways, it is reasonable to expect that cells attempt to downregulate genes that promote its transport.

## Discussion

In this study, we introduce a yeast-optimized table-top scRNA-seq platform for measuring single-cell level mRNA counts in yeast. The yeastDrop-Seq platform is applied to cells treated with Mycophenolic acid (MPA) and/or guanine, and the resulting changes in global mRNA expression levels were quantified. We elucidate heterogeneous gene expression profiles in cells as well as how the expression profiles are clustered in isogenic populations (Fig. 3). We further uncover differentially up- or downregulated groups of genes and pathways as a result of the growth conditions we used (Figs. 3–5). Our table-top scRNA-seq technology is similar in its function to a recently-published^6^ scRNA-seq technology that uses the commercial 10x Genomics platform; both technologies are droplet-based, involve microfluidics and adapted for yeast cells.

One major parameter to optimize for an effective yeastDrop-Seq is doublet formation rate, owing to the smaller size of *S. cerevisiae* cells (~4–5 μm in diameter) compared to mammalian cells (~10–100 µm). Although doublet-detection tools tailormade for yeast cells are yet not available, we assume transcription doublet in yeast cells share similar nature as that in mammalian cells. In this study, we applied five doublet-detection tools which are based on different algorithms to identify doublets in our dataset to estimate the doublet rate (Supplementary Fig. 4). Out of all tools, DoubletFinder^24^, scds^25^ Scrublet^26^, and solo^27^ have detected an average of 3.5% doublet rate and 2% of conserved doublets, doublets identified by more than one method used. Surprisingly, DoubletDecon^28^ has estimated an exceptionally high doublet rate with only 0.01% of the detected doublets is in agreement with the conserved doublets. This result could be explained by the benchmark experiment against DoubletFinder, Scrublet and solo; DoubletDecon tends to have higher sensitivity and lower specificity^28,29^. Therefore, we considered this inconsistent result as an outlier.

In our computational analysis, we found that the expression data from the cells grown in the 4 conditions could be merged without batch correction, suggesting that our protocol is highly reproducible with consistency. The principal component analysis (Fig. 2a) showed that the 4 samples are separated into 4 distinct clusters suggesting that each treatment condition had a distinct transcriptomic footprint. This result is further supported by our clustering results (Fig. 2d) as we could observe a similar cluster distribution over the 4 samples. Although we saw 4 distinct clusters in our UMAP analysis, we also observed subclusters (Fig. 2c) among the cells treated with DMSO, Guanine and MPA + Guanine, suggesting transcriptomic heterogeneity in these samples. This was an interesting observation as we used isogenic yeast cells across our experiments. To identify the subclusters, we performed unsupervised clustering in each condition and successfully identified 8 subclusters and their corresponding DE genes over the 4 conditions (Fig. 3). Collectively, our result reveals the population heterogeneity in *S. cerevisiae* in multiple conditions.

While higher levels of gene expression heterogeneity and cellular switching between gene expression states can facilitate an adaptive strategy against changing environmental conditions, it can be detrimental to population fitness in stable external conditions as decreasing the fraction of population at the optimal gene expression level would hamper fitness. Surprisingly, our results indicate that cells treated with MPA form a single subcluster, which is indicative of a transcriptionally homogeneous population. Future studies are needed to show how MPA’s lifespan-extension effect on yeast cells might be directly contributed by homogeneous expression of key lifespan-regulatory genes and how yeast cells aging in static environmental conditions could benefit more from the MPA treatment compared to cells aging in changing environments.

Although our current work focuses on the application of MPA on young yeast cells not enriched for old age, one can make predictions about how MPA extends lifespan based on the differential gene expression profiles we report here. For example, it is known that mitochondrial DNA accrues mutations as cells age, resulting in the accumulation of damaged mitochondria in the cell^23^. This leads to oxidative stress due to the mitochondrial malfunction and release of reactive oxygen species^30^. Forming a negative feedback regulation, the accumulation of reactive oxygen species can result in further damage in both mitochondrial and genomic DNA^31–33^. In this study, when yeast cells are treated with MPA, we see that, despite a downregulation of genes involved in mitochondrial function, there is an increase in the expression of genes coding for antioxidant proteins and response to reactive oxygen and decrease in the expression of genes coding for reactive oxygen species metabolic process (Supplementary Data 10). The upregulation of these genes could result in fewer reactive oxygen species and could therefore counter the effects of reactive oxygen species on cellular aging.

## Methods

### Cell growth conditions

Yeast cells with BY4741 genetic background (MATa his3Δ1 leu2Δ0 met15Δ0 ura3Δ0) were grown in 10 mL CSM minimal media containing 2% glucose. Four different treatment/growth conditions were used: minimal media containing (i) 10 µM DMSO (American Bio AB00435), (ii) 10 µM DMSO and 10 µM mycophenolic acid (Sigma M5255), (iii) 10 µM DMSO and 10 µM guanine (Sigma–Aldrich G11950) and 10 µM mycophenolic acid, (iv) 10 µM guanine. Cells were grown for 18 h to a final density (OD_600_) between 0.1 and 0.25. Cells were then diluted to 50 cells/μL for the final yeastDrop-Seq cell-collection using the microfluidic chip.

Doubling time of cells in each treatment condition was calculated as follows:

$${\mathrm{O{D}}}_{f}={\mathrm{O{D}}}_{i}\times \,{2}^{\frac{\Delta t}{{t}_{d}}}\to {\mathrm{lo{g}}}_{2}\frac{\mathrm{{O{D}}}_{f}}{\mathrm{{O{D}}}_{i}}=\frac{\Delta t}{{t}_{d}}\to {t}_{d}=\frac{\Delta t}{\mathrm{{lo{g}}}_{2}\frac{\mathrm{{O{D}}}_{f}}{\mathrm{{O{D}}}_{i}}}$$

where: $${\mathrm{O{D}}}_{f}={\mathrm{O{D}}}_{600}\,{\mathrm{final}}$$

OD_*i*_ = OD_600_ initial

$\Delta t$ = elapsed culture time (18 h)

${t}_{d}$ = calculated doubling time

Based on two-point cell density measurements, we found that the doubling time for MPA-treated cells was 187.8 min, for DMSO-treated cells was 130 min, for guanine-treated cells was 130 min, and for GM-treated cells was 158.9 min.

### yeastDrop-Seq experiments

Cells were subjected to Drop-Seq following the original protocol^2^ with the following modifications. Barcoded beads were resuspended in the yeastDrop-Seq solution containing the reagents as shown in Table 1. Cells were diluted to 50 cells/μL. After initial droplet formation, droplets were incubated for 30 min at 30 °C to ensure zymolyase breakdown of cell walls followed by cell bursting (Supplementary Fig. 1). After incubation, droplet quality was evaluated and >95% droplet-uniformity remained intact.

**Table 1:** yeastDrop-Seq solution.

Solution (total volume)	Reagent	Reagent volume
yeastDrop-seq solution (2223.5 μL)	Zymolyase (Zymo Research E1004), 2 Units/μL	37.5 μL
	Lysis buffer (no DTT)	1.5 mL
	1 M DTT	75 μL
	Zymolyase buffer	600 μL
	10% SDS (final concentration at 0.05%)	11 μL
Lysis buffer (no DTT)^2^ (950 μL)	H_2_O	500 μL
	20% Ficoll PM-100	300 μL
	Sarkosyl	10 μL
	0.5 M EDTA	40 μL
	2 M Tris pH 7.5	100 μL
Zymolyase buffer^37^ (CSH protocols Zymolyase Buffer) (100 mL)	2 M Sorbitol	50 mL
	1 M K_2_HPO_4_	4.2 mL
	1 M KH_2_PO_4_	0.8 mL
	EDTA (0.5 M, pH 8)	1 mL
	H_2_O	44 mL

Downstream breakage of oil droplets followed by reverse transcription, exonuclease I treatment, and PCR for cDNA amplification were carried out as per the Drop-Seq protocol^2^. cDNA was then tagmented using the NEBNext Ultra II DNA Library Prep with Sample Purification Beads kit (NEB #E7103S).

Sequencing was done using the Illumina HiSeq2500 platform with 2 × 100 read pairs.

### Single-cell RNA-seq data processing

Sequencing reads were trimmed using Trimmomatic (version 0.39) for adapters and any base with less than 30 quality score was also removed. Reads were aligned to the *S. cerevisiae* reference genome (Genome assembly: R64-1-1 (GCA_000146045.2), ensembl genomes) using STAR (version 2.7.5a). The expression count matrix was generated using UMI-tools (version 1.0.1) on Saccharomyces_cerevisiae.R64-1-1.102 transcript annotation. Quality control was performed using the scater R package (version 1.12.2). Number of transcripts counts (UMI) in cells and number of features (genes) of each sample were fitted to the Gaussian distribution and the outliers were filtered. Refer to the code availability section for the code and parameters.

### SC3 clustering

The cells of the 4 samples were combined and normalized using the command ‘NormalizeData()‘ from the Seurat R package (version 3.2.0). The PCA plot was performed using the top 500 variable features. Sample heterogeneity was analysed using the SC3 R package (version 1.15.1). The number of clusters in each sample was evaluated by assessing the heatmap of consensus clustering and the silhouette plot of clusters. The map of cells from samples to the SC3 determined clusters was made as a Sankey plot from the network3D R package (version 0.4).

### Identification of differentially expressed genes

Differentially expressed genes in each sample and consensus clusters k = 4, k = 8 were analysed using the Seurat R package (version 3.2.0). All samples are combined and log normalized with a scale factor of 10^4^. The Wilcoxon test was used for statistical tests, and the Bonferroni correction method was used to adjust the *p*-values of each gene. The key DE genes in Figs. 4 and 5 are defined by a threshold of 50% minimum percentage of cells with the gene detected and 0.5 log2 fold change was used to filter the gene list.

### Doublet rate analysis

Doublet analyses were performed over 5 tools, including DoubletFinder (version 2.0.3), DoubletDecon (version 1.1.6), scds (version 1.1.2) R packages, Scrublet (version 0.2.1) and solo (version 0.6) python packages. For DoubletFinder, the value of pK in each sample was determined by the mean-variance normalized bimodality coefficient. For DoubletDecon, the rhop value was set to 0.8. For scds, the threshold for doublet was determined by the co-expression based doublet scores, binary classification based doublet scores, and the hybrid scores. A threshold of top 4% cells was set for each score. For Scrublet, the transcriptomic doublet was determined by comparing to the doublet scores of simulated doublets in each sample. For solo, the default parameters were used. For the cells that were flagged as doublets in more than one of the tools (excluding DoubletDecon due to inconsistency), were considered as high-confidence doublets.

### GO term analysis

Each cluster had genes that were classified as up- or downregulated based on fold change in expression and statistical significance with p-value less than 0.05, using the Seurat (version 3). The R package clusterProfiler [ref] was then used to perform the GO term enrichment and pathway identifications. In brief, the ‘enrichGO()‘ function was used with the org.Sc.sgd.db organism database and the Biological Process of the GO term database. The reported *p*-value is further adjusted using the Benjamini–Hochberg procedure to correct for multiple testing^34^.

### Statistics and reproducibility

The information on how statistical tests were conducted is provided in the relevant subsections of the Methods section. For example, the Wilcoxon test was used as the statistical test associated with the identification of differentially expressed genes, with the use of the Bonferroni correction method to adjust the *p*-values of each gene. The sample size was targeted based on the original (mammalian cell optimized) Drop-Seq technique^2^. Corresponding n number of each sample or cluster is indicated where appropriate. Due to the inherent nature of stochasticity associated with gene expression, there were cell-to-cell differences in the analyzed RNA transcript numbers even for cells grown in the same growth condition.

### Reporting summary

Further information on research design is available in the Nature Research Reporting Summary linked to this article.

## Supplementary information

Supplementary Information

Description of Additional Supplementary Files

Supplementary Data 1

Supplementary Data 2

Supplementary Data 3

Supplementary Data 4

Supplementary Data 5

Supplementary Data 6

Supplementary Data 7

Supplementary Data 8

Supplementary Data 9

Supplementary Data 10

Reporting Summary

## Data Availability

The accession number for the raw sequencing data corresponding to the four treatment conditions is GEO: GSE165686. Source data used as inputs to generate the figure panels, with each Excel file containing the data shown in specific figure panel(s) are available at the DOI-minting repository Zenodo at 10.5281/zenodo.4767298^35^. All other data are available from the corresponding authors on reasonable request.

## Code availability

The code for analyses and parameters are available at https://github.com/hemberg-lab/yeastDrop-Seq and the DOI-minting repository Zenodo at 10.5281/zenodo.4762526^36^.
