# Author Correction: A yeast-optimized single-cell transcriptomics platform elucidates how mycophenolic acid and guanine alter global mRNA levels

**DOI:** 10.1038/s42003-021-02420-7

**Published:** 2021-07-13

**Authors:** Guste Urbonaite, Jimmy Tsz Hang Lee, Ping Liu, Guillermo E. Parada, Martin Hemberg, Murat Acar

**Affiliations:** 1 Systems Biology Institute, Yale University, West Haven, CT USA; 2 Department of Molecular Cellular and Developmental Biology, Yale University, New Haven, CT USA; 3 Wellcome Sanger Institute, Wellcome Genome Campus, Hinxton, UK; 4 Department of Physics, Yale University, New Haven, CT USA; 5 Present Address: Evergrande Center for Immunologic Disease, Harvard Medical School and Brigham and Women’s Hospital, Boston, MA USA

**Keywords:** Gene expression analysis, Saccharomyces cerevisiae, Biochemical networks, Genetic interaction

Correction to: *Communications Biology* 10.1038/s42003-021-02320-w, published online 30 June 2021.

In the original version of the Article, reference 29 contained errors in the journal name, page numbers, and second author’s initials. The incorrect reference shown was:

Xi, N. M. & Li, J. Y. Benchmarking computational doublet-detection methods for single-cell RNA sequencing data. *Sci. Direct* **12**, 1–19 (2020).

The correct reference is:

Xi, N. M. & Li, J. J. Benchmarking Computational Doublet-Detection Methods for Single-Cell RNA Sequencing Data. *Cell Syst*. **12**, 176–194.e6 (2021).

In addition, the page numbers provided in references 15 and 16 were incorrectly given as 316689 – 331700 and 294 – 319, respectively. The correct page numbers are 31689-31700 (reference 15) and e00294-19 (reference 16).

The errors have been corrected in the HTML and PDF versions of the article.

