# Supplementary material for: A yeast-optimized single-cell transcriptomics platform elucidates how mycophenolic acid and guanine alter global mRNA levels
Source: Commun Biol. 2021 Jun 30;4:822. doi: 10.1038/s42003-021-02320-w (PMC8245502; doi:10.1038/s42003-021-02320-w)
Supplement: Supplementary file 1 — Supplementary Information [file 42003_2021_2320_MOESM1_ESM.pdf]

SUPPLEMENTARY INFORMATION

Supplementary Figures

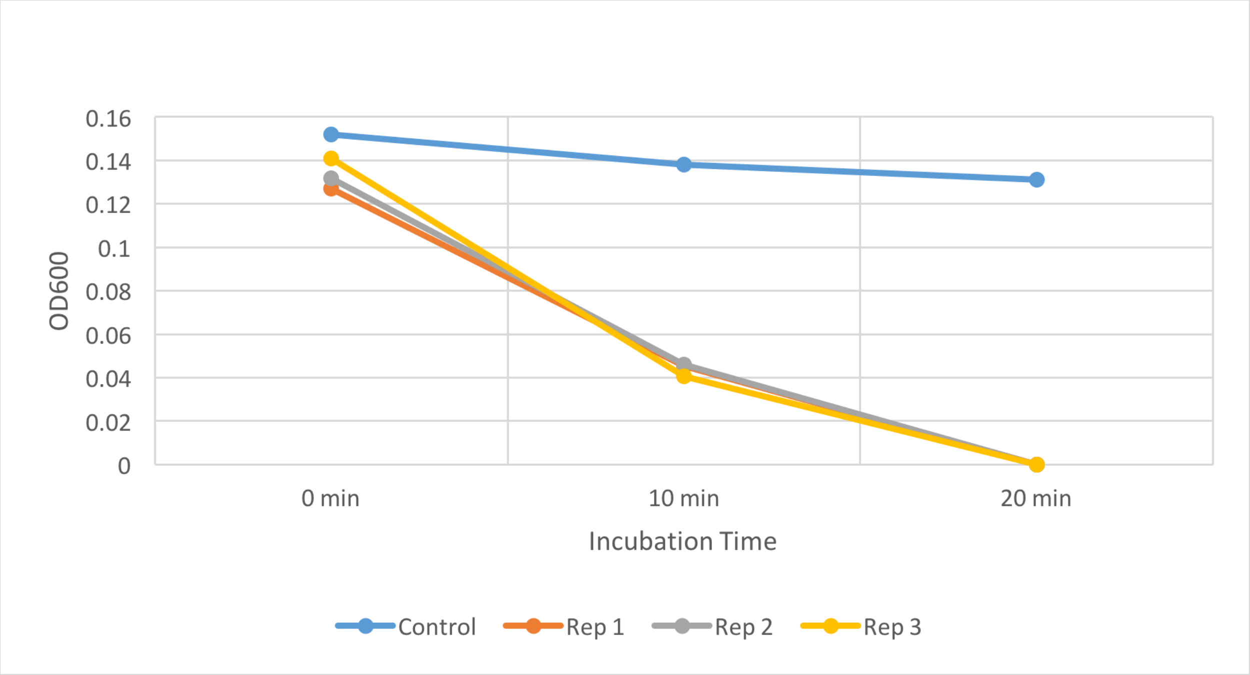

Supplementary Figure 1. Cell burst dynamics. Cells were grown overnight in CSM media with 2% glucose to a density of 0.51 (OD_{600}). Then, cells were either incubated in yeastDrop-Seq solution (Replicates 1-3) or CSM media (control). OD_{600} values were measured every 10 minutes to determine the concentration of intact cells. After 20 minutes, all three replicates had an $OD_{600}$of approximately 0.

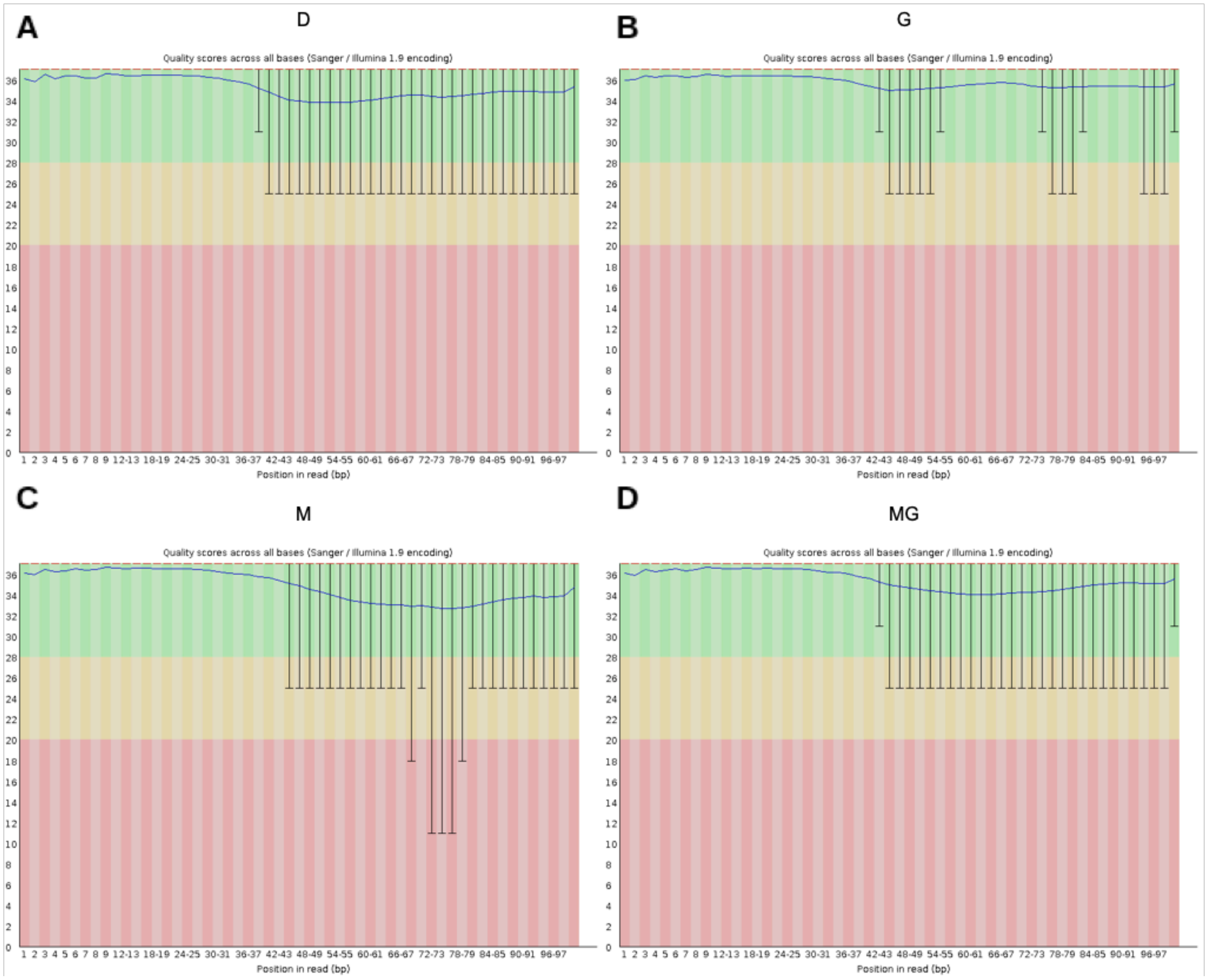

Supplementary Figure 2. FASTQ results of the 4 samples. Plots of PHRED 33 scores of the raw reads of A) DMSO, B) Guanine, C) MPA and D) MPA+Guanine (MG) samples. For each position a Box plot is drawn. The upper and lower whiskers represent the 10% and 90% points. The blue line represents the mean quality. The y-axis on the graph shows the quality scores. The background of the graph divides the y-axis into very good quality calls (green), calls of reasonable quality (orange), and calls of poor quality (red).

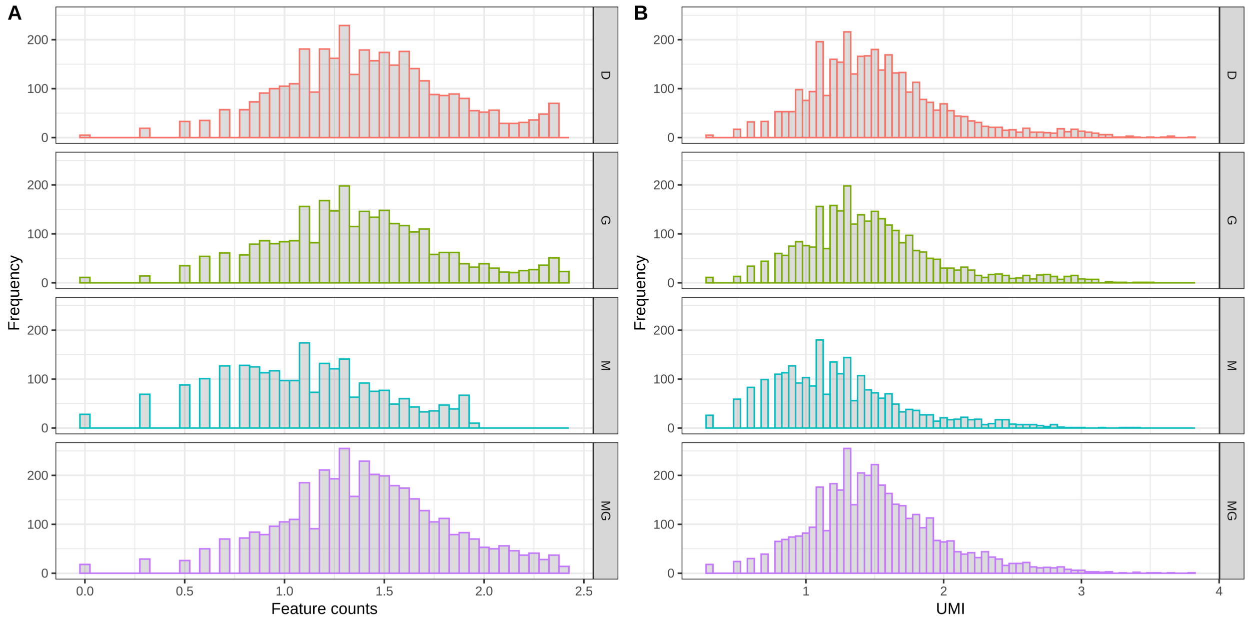

Supplementary Figure 3. Plots of frequencies of A) feature counts and B) UMI per cell in the 4 samples (D n=233, G n=258, M n=85, MG n=268 cells).

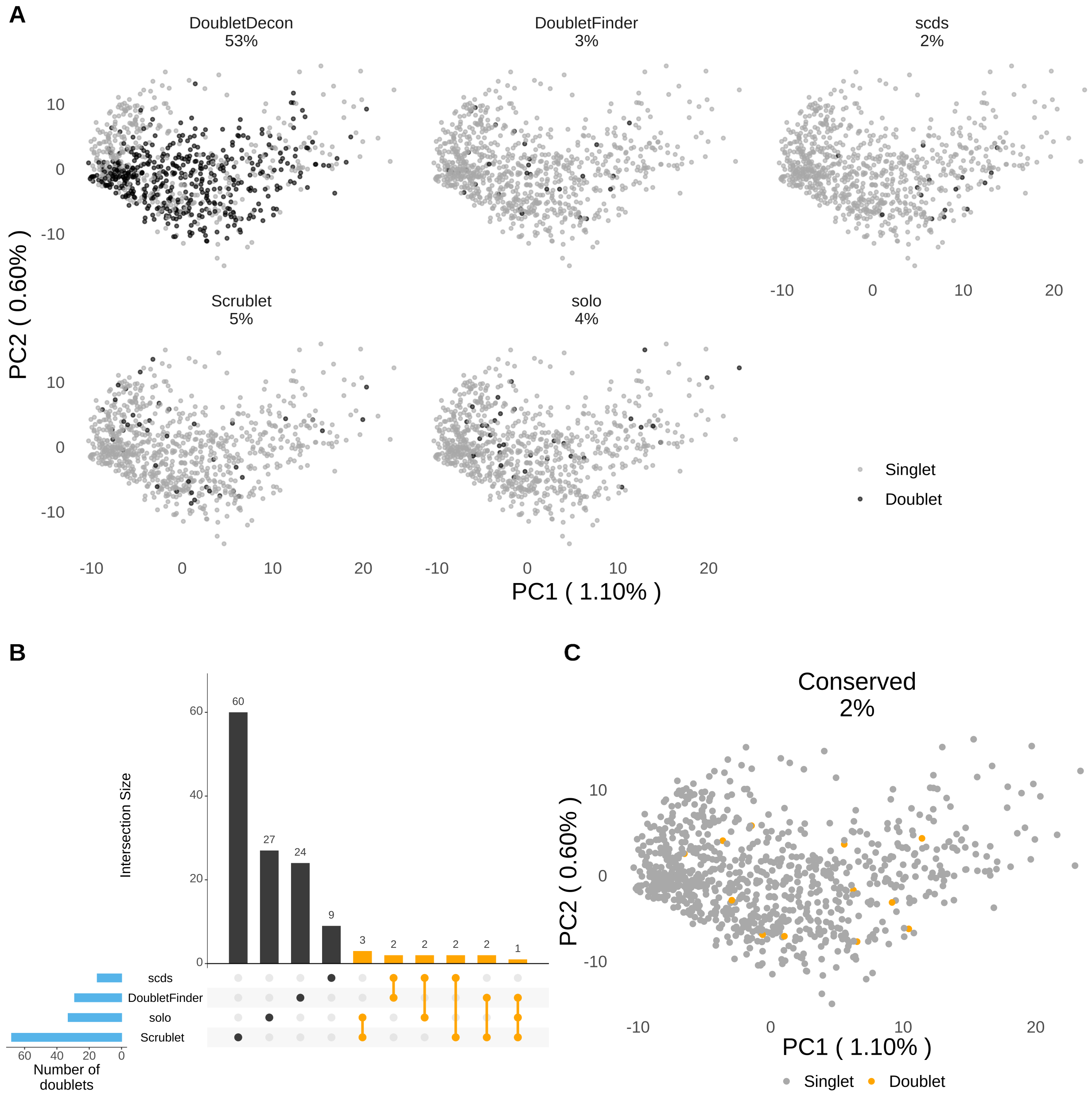

Supplementary Figure 4. Doublet rate analyses over 5 tools. A) PCA plots showing the cells that are flagged as predicted doublets by using DoubletDecon, DoubletFinder, scds, Scrublet and solo. The percentage of doublet on top of each PCA

plot is calculated by the number of predicted doublets in all cells across the 4 samples. The denominator used in percentage determination is the total number of all cells across the 4 samples (n=844 cells). B) Upset plot showing the overlap of 132 doublets predicted by DoubletFinder, Scrublet, scds and solo. The horizontal bar chart (blue) is representing the number of doublets predicted in the corresponding tool. The intersection size is representing the number of identical doublets predicted in corresponding tools. For doublets found by more than one tool (conserved doublet), the corresponding bar is highlighted in yellow C) PCA plot showing the cells that were flagged as doublets in more than one of the tools. The points highlighted in yellow are the doublets corresponding to the yellow bars in the Upset plot of panel B.

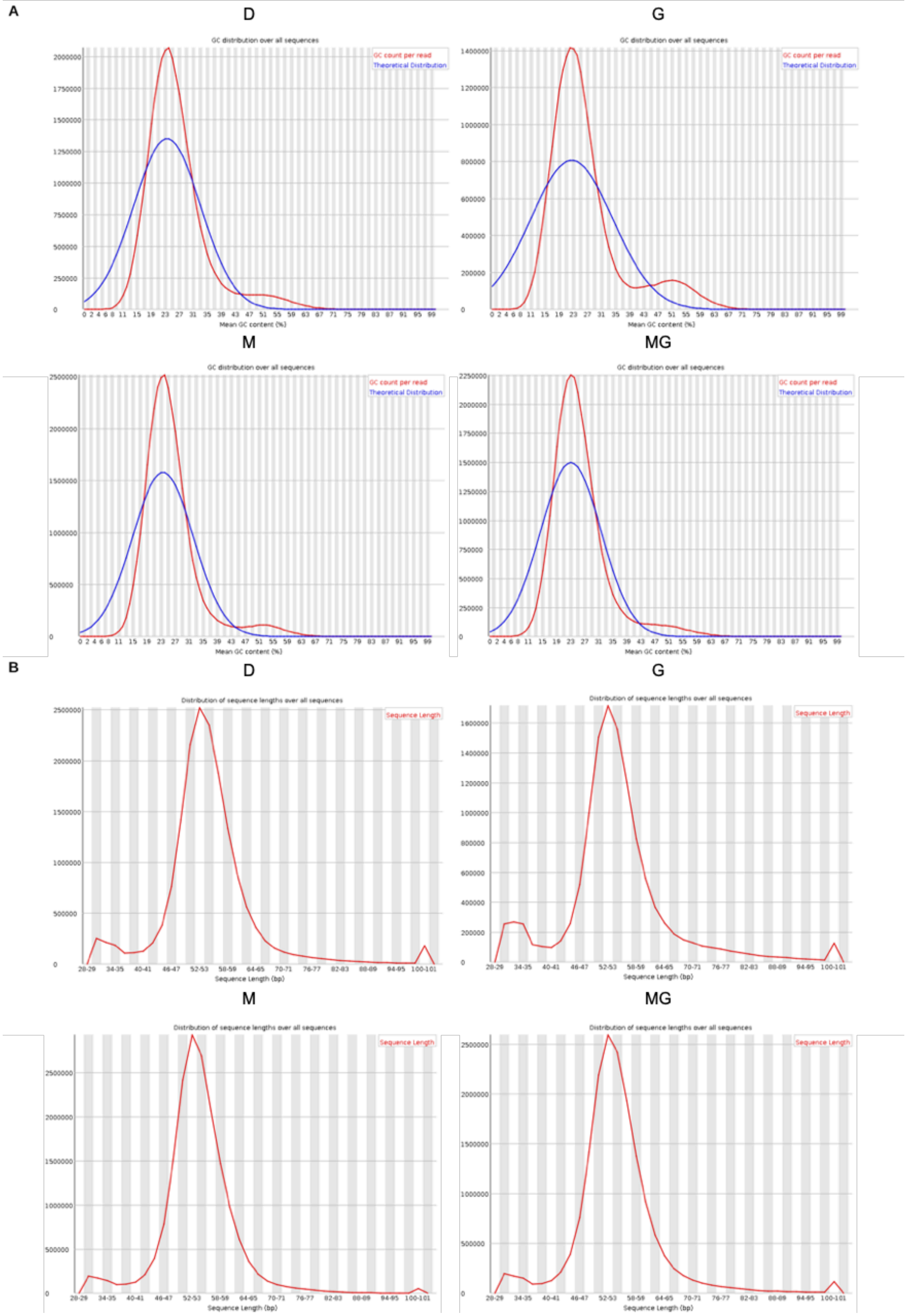
A
D
G
M
MG
GC distribution over all sequences
GC count per read
Theoretical Distribution
Mean GC content (%)
B
D
G
M
MG
Distribution of sequence lengths over all sequences
Sequence Length
Sequence Length (bp)

Supplementary Figure 5. FASTQ results of the 4 samples. A) Plot of percentage of GC content in the 4 samples. The GC content across the whole length of each raw read sequence is the denominator of the calculations. B) Plot of overall sequence length of the 4 samples after adaptor trimming process.

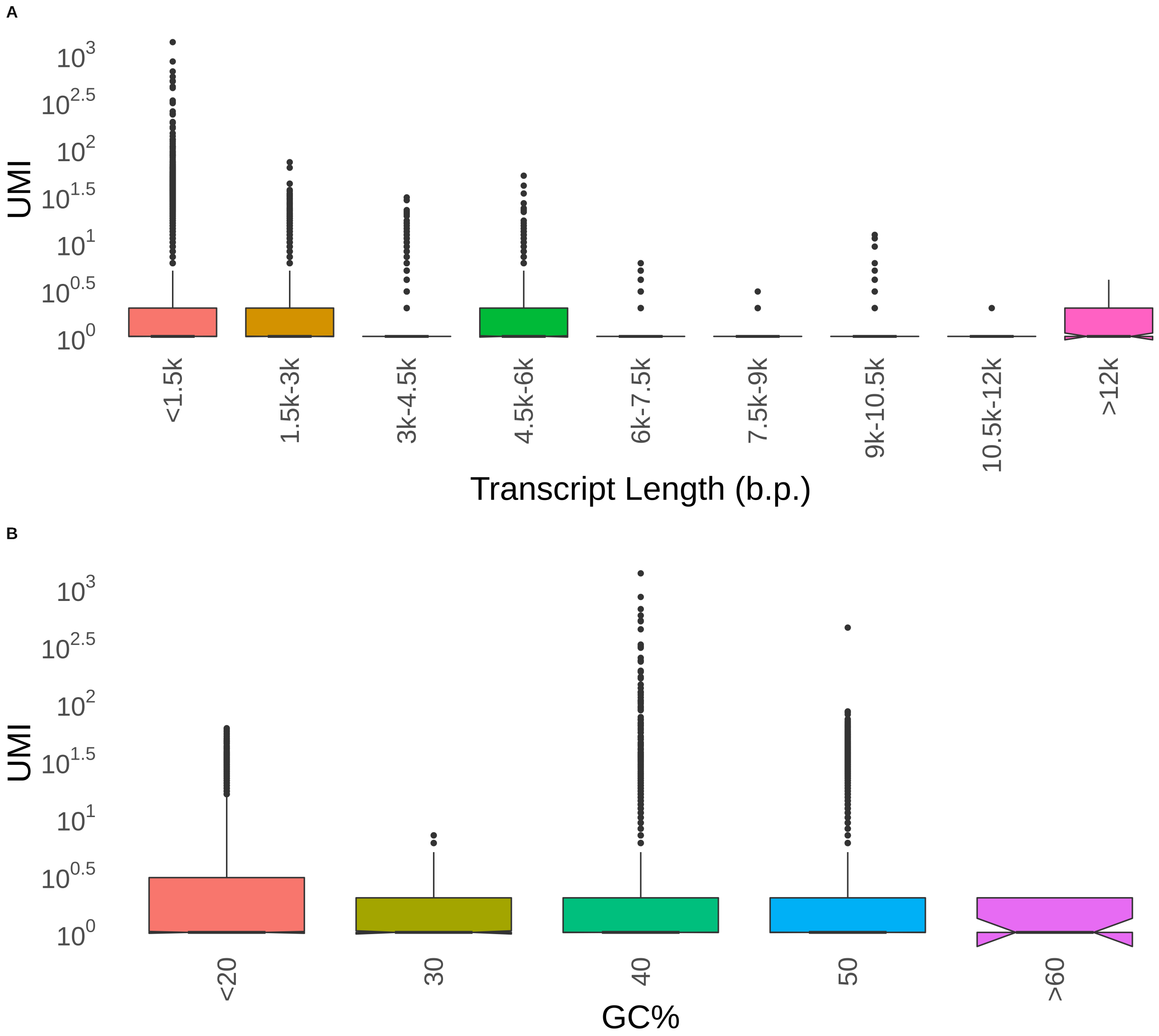

Supplementary Figure 6. Distribution of UMI counts over S. cerevisiae A) transcript length and B) percentage of GC content of transcripts. The GC content

across the whole length of corresponding transcript is the denominator of the calculations. Box plots show the median (center line), interquartile range (hinges) and 1.5 times the interquartile range (whiskers); outlier data beyond this range are plotted as individual points.

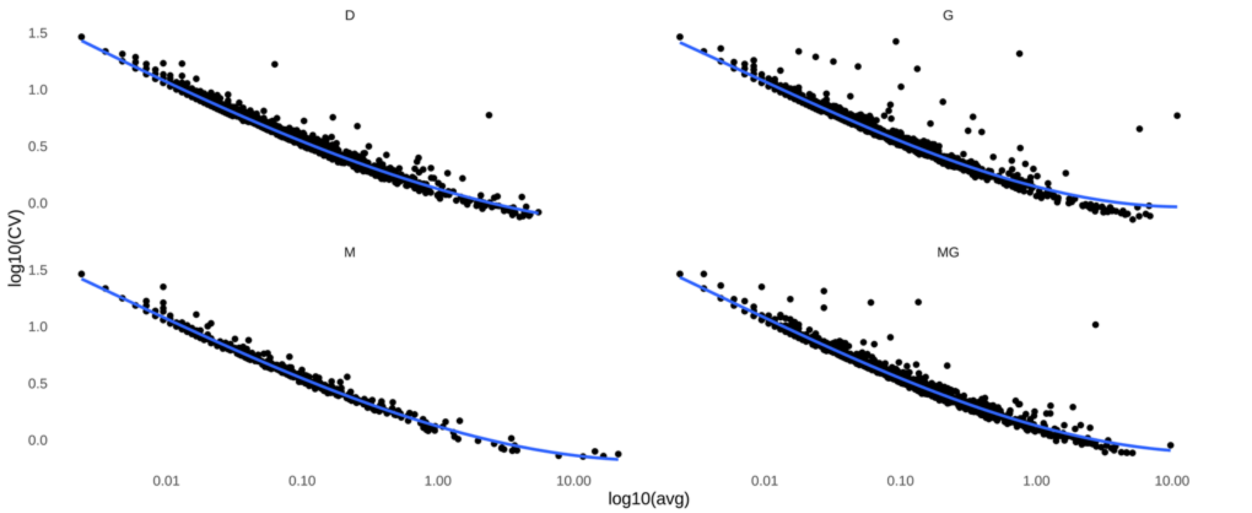

Supplementary Figure 7. Plots of coefficient of variations of mean transcript numbers. The mean is calculated as the averaged expression levels of each gene of the corresponding sample (D n=233, G n=258, M n=85, MG n=268 cells). Blue line is fitted by a second-degree polynomial (formula = y~poly(x, 2) in the function geom_smooth() of ggplot2).
