# Supplementary material for: A yeast-optimized single-cell transcriptomics platform elucidates how mycophenolic acid and guanine alter global mRNA levels
Source: Commun Biol. 2021 Jun 30;4:822. doi: 10.1038/s42003-021-02320-w (PMC8245502; doi:10.1038/s42003-021-02320-w)
Supplement: Supplementary file 2 — Description of Additional Supplementary Files [file 42003_2021_2320_MOESM2_ESM.pdf]

Description of Additional Supplementary Files

File Name: Supplementary Data 1
Description: Distribution of the number of distinct mRNA transcripts per cell for each treatment condition.

File Name: Supplementary Data 2
Description: Coefficient of variations (CV) of mean transcript numbers.

File Name: Supplementary Data 3
Description: The data of the heatmap for DE genes corresponding to the 4 samples; each number corresponds to the normalized expression level in a single cell.

File Name: Supplementary Data 4
Description: List of marker genes corresponding to each subcluster from the 4 treatment conditions identified by AUROC.

File Name: Supplementary Data 5
Description: The data of the Heatmap for DE genes corresponding to the 8 sub-clusters identified by SC3; each number corresponds to the normalized expression level in a single cell.

File Name: Supplementary Data 6
Description: List of marker genes corresponding to the 4 treatment conditions identified by Wilcoxon test.

File Name: Supplementary Data 7
Description: List of marker genes corresponding to the subclusters of the 4 treatment conditions identified by Wilcoxon Test.

File Name: Supplementary Data 8
Description: List of marker genes corresponding to the SC3 clusters identified by Wilcoxon test.

File Name: Supplementary Data 9
Description: Gene ontology analysis of the 4 conditions.

File Name: Supplementary Data 10
Description: Gene ontology analysis of the subclusters corresponding to 4 conditions.
