# Supplementary material for: A yeast-optimized single-cell transcriptomics platform elucidates how mycophenolic acid and guanine alter global mRNA levels
Source: Commun Biol. 2021 Jun 30;4:822. doi: 10.1038/s42003-021-02320-w (PMC8245502; doi:10.1038/s42003-021-02320-w)
Supplement: Supplementary file 13 — Reporting Summary [file 42003_2021_2320_MOESM13_ESM.pdf]

nature research

Corresponding author(s): Murat Acar and Martin Hemberg

Last updated by author(s): 5/11/2021

Reporting Summary

Nature Research wishes to improve the reproducibility of the work that we publish. This form provides structure for consistency and transparency in reporting. For further information on Nature Research policies, see our Editorial Policies and the Editorial Policy Checklist.

Statistics

For all statistical analyses, confirm that the following items are present in the figure legend, table legend, main text, or Methods section.

n/a	Confirmed	
☐	☒	The exact sample size (n) for each experimental group/condition, given as a discrete number and unit of measurement
☐	☒	A statement on whether measurements were taken from distinct samples or whether the same sample was measured repeatedly
☐	☒	The statistical test(s) used AND whether they are one- or two-sided<br>*Only common tests should be described solely by name; describe more complex techniques in the Methods section.*
☒	☐	A description of all covariates tested
☒	☐	A description of any assumptions or corrections, such as tests of normality and adjustment for multiple comparisons
☐	☒	A full description of the statistical parameters including central tendency (e.g. means) or other basic estimates (e.g. regression coefficient) AND variation (e.g. standard deviation) or associated estimates of uncertainty (e.g. confidence intervals)
☒	☐	For null hypothesis testing, the test statistic (e.g. F, t, r) with confidence intervals, effect sizes, degrees of freedom and P value noted<br>*Give P values as exact values whenever suitable.*
☒	☐	For Bayesian analysis, information on the choice of priors and Markov chain Monte Carlo settings
☒	☐	For hierarchical and complex designs, identification of the appropriate level for tests and full reporting of outcomes
☒	☐	Estimates of effect sizes (e.g. Cohen's d, Pearson's r), indicating how they were calculated

Our web collection on statistics for biologists contains articles on many of the points above.

Software and code

Policy information about availability of computer code

Data collection	RNA sequencing was performed using the Illumina HiSeq2500 platform with 2x100 read pairs.
Data analysis	The details of data analysis and the software/code used are provided in the relevant subsections of the Methods section. Also, the code for analyses and parameters are available at https://github.com/hemberg-lab/yeastDrop-Seq.

For manuscripts utilizing custom algorithms or software that are central to the research but not yet described in published literature, software must be made available to editors and reviewers. We strongly encourage code deposition in a community repository (e.g. GitHub). See the Nature Research guidelines for submitting code & software for further information.

Data

Policy information about availability of data

All manuscripts must include a data availability statement. This statement should provide the following information, where applicable:

- Accession codes, unique identifiers, or web links for publicly available datasets
- A list of figures that have associated raw data
- A description of any restrictions on data availability

The accession number for the raw sequencing data corresponding to the four treatment conditions is GEO: GSE165686.

Field-specific reporting

Life sciences study design

All studies must disclose on these points even when the disclosure is negative.

Sample size	We targeted a sample size based on the original (mammalian cell optimized) Drop-Seq technique (Macosko E. et al, Cell, 2015).
Data exclusions	No data were excluded.
Replication	Due to the inherent nature of stochasticity associated with gene expression, there were cell-to-cell differences (as expected) in the analyzed RNA transcript numbers even for cells grown in the same growth condition.
Randomization	N/A (no randomization)
Blinding	N/A (no blinding)

Reporting for specific materials, systems and methods

We require information from authors about some types of materials, experimental systems and methods used in many studies. Here, indicate whether each material, system or method listed is relevant to your study. If you are not sure if a list item applies to your research, read the appropriate section before selecting a response.

Materials & experimental systems

n/a	Involved in the study
☒	☐ Antibodies
☐	☒ Eukaryotic cell lines
☒	☐ Palaeontology and archaeology
☒	☐ Animals and other organisms
☒	☐ Human research participants
☒	☐ Clinical data
☒	☐ Dual use research of concern

Methods

n/a	Involved in the study
☒	☐ ChIP-seq
☒	☐ Flow cytometry
☒	☐ MRI-based neuroimaging

Eukaryotic cell lines

Policy information about cell lines

Cell line source(s)	Saccharomyces cerevisiae (Acar Lab)
Authentication	Validated its appropriate auxotrophy on plates lacking the appropriate aminoacids
Mycoplasma contamination	N/A
Commonly misidentified lines (See ICLAC register)	N/A
